# A randomized phase III study of the docetaxel/carboplatin combination versus docetaxel single-agent as second line treatment for patients with advanced/metastatic Non-Small Cell Lung Cancer

**DOI:** 10.1186/1471-2407-10-633

**Published:** 2010-11-19

**Authors:** Athanasios G Pallis, Sophia Agelaki, Athina Agelidou, Ioannis Varthalitis, Kostas Syrigos, Nikolaos Kentepozidis, Georgia Pavlakou, Athanasios Kotsakis, Emmanouel Kontopodis, Vassilis Georgoulias

**Affiliations:** 1 Hellenic Oncology Research Group (HORG), 55 Lombardou str., 114 74 Athens, Greece; 2 Department of Medical Oncology, University General Hospital of Heraklion, P.O. Box: 1352, Heraklion 71110, Crete, Greece

## Abstract

**Background:**

To compare the activity and toxicity of docetaxel/carboplatin (DC) doublet vs single agent docetaxel (D) as second-line treatment in patients with advanced non-small cell lung cancer (NSCLC).

**Methods:**

Patients pre-treated with front-line platinum-free regimens, were randomized to receive either docetaxel/carboplatin (DC), (docetaxel 50 mg/m^2^; carboplatin AUC4; both drugs administered on days 1 and 15) or docetaxel single-agent (D), (docetaxel 50 mg/m^2 ^on days 1 and 15).

**Results:**

Response rate was similar between the two arms (DC vs D: 10.4% vs 7.7%; p = 0.764). After a median follow-up time of 28.0 months for DC arm and 34.5 months for D arm, progression free survival (PFS) was significantly higher in the DC arm (DC vs D:3.33 months vs 2.60 months; p-value = 0.012), while no significant difference was observed in terms of overall survival (OS) (DC vs D: 10.3 months vs 7.70 months; p-value = 0.550). Chemotherapy was well-tolerated and grade III/IV toxicities were relatively infrequent. No toxic deaths were observed.

**Conclusions:**

This study has not achieved its primary objective of significant OS prolongation with docetaxel/carboplatin combination over single-agent docetaxel in patients who had not received front-line docetaxel; however, the docetaxel/carboplatin combination was associated with a significant clinical benefit in terms of PFS.

## Background

Lung cancer represents a major health problem world-wide. It is the leading cause of cancer-related death in Europe [1], with a 5-year survival of approximately 15% for all stages [2]. Non-Small Cell Lung Cancer (NSCLC) represents approximately 80-85% of all lung carcinomas. The vast majority of patients are diagnosed with advanced, unresectable disease (stage IIIB/IV) which remains incurable with a 5-year survival rate of less than 5% [3].

Front-line chemotherapy for the treatment of patients with inoperable locally advanced or/and metastatic NSCLC has been substantially improved during the last decade with the introduction of new cytotoxic agents such as gemcitabine, vinorelbine, gemcitabine, paclitaxel, docetaxel and pemetrexed; combination of these agents with a platinum compound represents the standard of care for front-line treatment [4]. Nevertheless, several individual phase III trials have demonstrated that there is no difference between platinum-based and platinum-free doublets in terms of overall response rate (ORR), time to tumor progression (TTP) and overall survival (OS), while platinum-free doublets have a more favorable toxicity profile [5-9]. However, NSCLC patients will inevitably experience tumor progression and at that time some patients will still have a good Performance Status (PS) and will be suitable for second-line treatment.

Two phase III trials have demonstrated that administration of docetaxel in the second-line setting was superior in terms of OS compared to best supportive care [10] and improved TTP and 1-year survival rate compared to vinorelbine or ifosfamide [11]. Furthermore, the administration of pemetrexed in the second-line setting was associated with an OS comparable to that of docetaxel with a more favorable toxicity profile [12].

The epidermal growth factor receptor (EGFR) tyrosine kinase inhibitors (TKIs) erlotinib and gefitinib are also considered as an alternative for second-line treatment since erlotinib was associated with a superior OS compared to placebo [13] whereas gefitinib was associated with a comparable OS when compared to docetaxel [14]. However, results of second-line treatment for patients with relapsing or progressing disease are generally poor, with response rate of less than 10% and OS of 7-8 months [10-14]. One logical approach to improve these results is to evaluate combination regimens. Two phase II trials have evaluated the docetaxel/carboplatin combination as second-line treatment in NSCLC demonstrating encouraging results with an ORR of ≥20% and a median OS of 8 months [15,16]. However, this combination has not been compared against the standard second line treatment with single agent docetaxel. Furthermore, given that there is no clear evidence that platinum-based doublets offer a survival benefit against third-generation platinum-free regimens [17] there will be a number of NSCLC patients treated with platinum-free combinations as first line treatment and for whom a platinum-based doublet as second line treatment will be a logical approach. These observations provided the rationale for this phase III trial which was conducted in order to determine whether the combination of docetaxel/carboplatin provides any therapeutic benefit compared to single-agent docetaxel.

## Methods

### Patients

Eligible patients were aged 18 years or older, with histologically or cytologically confirmed, unresectable locally advanced (stage IIIB with pleural or pericardial effusion) or metastatic (stage IV) NSCLC, that progressed or recurred after one previous platinum-free and docetaxel-free chemotherapy regimen. Patients had to have a life expectancy of more than 3 months and a World Health Organization (WHO) performance status (PS) of ≤ 2, and adequate organ function [serum bilirubin ≤ 1.5 times the upper normal limit (UNL); AST and ALT ≤ 2.5 UNL in the absence of perceptible liver metastases, or ≤ 5 UNL in the presence of liver metastases; serum creatinine ≤ 1.5 times the UNL; neutrophils ≥ 1.5 × 10-9/L, and platelets ≥ 100 × 10-9/L]. Patients with known, symptomatic central nervous system metastases were ineligible. Other eligibility criteria were: absence of active infection, history of significant cardiac disease (unstable angina, congestive heart failure, myocardial infarction within the previous 6 months, ventricular arrhythmias) or malnutrition (loss of ≥ 20% of the original body weight). All patients gave written informed consent to participate in the study and the trial was approved by the Ethics and Scientific Committees of the participating centers. The study was conducted according to the Helsinki Declaration and Good Clinical Practice guidelines.

### Treatment

For the docetaxel/carboplatin arm (DC), treatment consisted of docetaxel at a dose of 50 mg/m^2^, administered as a 60-min iv infusion on days 1 and 15; carboplatin was administered on days 1 and 15 as a 90-min intravenous infusion, at a dose of AUC 4. Standard pre- and post-medication with 8 mg oral dexamethasone, at 7 h and 1 h before the docetaxel infusion, and 8 mg twice daily for a further 3 days, was used to reduce the risk of allergic reactions and fluid-retention syndrome that can be associated with docetaxel administration. For the docetaxel single-agent arm (D), treatment consisted of docetaxel at a dose of 50 mg/m^2 ^on days 1 and 15 after appropriate corticosteroid pre- and post-medication. The every two weeks schedule was selected because of its more favorable toxicity profile [18-20]. Treatment was repeated every four weeks, for both arms. Treatment was continued until disease progression, the appearance of unacceptable toxicity, or patient's withdrawal of consent, for a maximum of 6 cycles.

### Βaseline and follow-up assessments

Baseline assessment included a complete medical history, evaluation of performance status, physical examination and vital signs, 12-lead ECG, blood tests (complete blood cell count with differential and blood chemistry), chest X-rays and computed tomography scans of the chest, abdomen and brain and a whole-body radionuclide bone scan. Baseline evaluation had to be performed within two weeks prior to therapy initiation. All measurable lesions were identified at baseline and were monitored throughout. A complete medical history and a detailed physical examination with complete blood cell count with differential and blood chemistry, ECG and a chest X-ray were performed before each treatment administration to assess the disease status and treatment toxicity. After completion of study treatment, patients were followed every month until the development of disease progression. Third-line therapy included best supportive care, palliative radiotherapy, or chemotherapy according to the discretion of the responsible physician.

### Assessment of antitumor activity and toxicity

Response assessment was evaluated every two chemotherapy cycles, and every one month after treatment completion. Objective tumor responses were evaluated according to RECIST criteria [21]. All CT scans were reviewed by an independent radiologist. Toxicity was evaluated before each chemotherapy administration and was reported according to National Cancer Institute Common Toxicity Criteria, version 2 [22].

### Statistical analysis

Patients were centrally randomized by computer software to a 1:1 ratio, to receive either DC or D. The randomization to each arm was done by stratification according to PS, stage of disease and response to front-line treatment.

The primary end-point was overall survival (OS); secondary endpoints included overall response rate (ORR), safety profile and progression-free survival (PFS) associated with each regimen. The study was designed to have 90% power (α = 0.05, two-sided log-rank test) to detect an increase in median survival from 4 months for the single agent docetaxel arm [18] to 8 months for the docetaxel/carboplatin arm [15,16] at the statistically significant level of 5%. Sixty-five patients should be enrolled in each arm to achieve the statistical requirements.

Analysis was performed on an intent-to-treat basis. Duration of tumor response is measured from the date the first objective response (complete or partial) was observed to the first date of tumor progression or death from any cause. The PFS was measured from study entry until the day of the first evidence of disease progression whereas OS from the date of study entry to death or last contact. The probability of survival was calculated by the method of Kaplan-Meier [23] and tested for differences by using the log-rank test. All tests were two-sided and were considered significant when the resulting p-value was ≤ 0.05. This study is registered with ClinicalTrials.gov, number NCT00430651.

## Results

### Patient demographics

From 08/2004 until 03/2008, 67 patients were enrolled in the DC arm and 65 in the D arm. Twelve (17.9%) patients completed treatment as per protocol in the DC arm and 9 (13.8%) patients in the D arm (p-value = 0.636). Early treatment discontinuation before the administration of 6 chemotherapy cycles, because of disease progression, occurred in 49 (73.1%) and 51 (78.5%) patients in the DC and the D arm, respectively. Four (6.0%) and two (3.1%) patients in the DC and D arms, respectively discontinued treatment due to adverse events (p-value = 0.680). These patients were considered to have progressive disease in the intention-to-treat analysis. Patients' characteristics are presented in Table 1.

**Table 1:** Patients Characteristics

	**DC** **(n = 67)**	**D** **(n = 65)**	
		
	**N (%)**	**N (%)**	**P-value**
	
Age	62	63	
*Median (min-max)*	39 - 78	39 - 83	
Sex			
*Male*	64 (95.5)	56 (86.2)	P = 0.074
*Female*	3 (4.5)	9 (13.8)	
Performance status			
*0*	26 (38.8)	14 (21.5)	P = 0.088
1	33 (49.3)	39 (60.0)	
*2*	8 (11.9)	12 (18.5)	
Histological Type			
*Squamous*	16 (23.9)	18 (27.7)	Adeno Ca vs Non-Adeno Ca P = 0.380
*Adeno Ca*	26 (38.8)	31 (47.7)	
*Large Cell*	2 (3.0)	1 (1.5)	
*Mixed*	1 (1.5)	1 (1.5)	
*Other*	22 (32.8)	14 (21.5)	
No. of Organs Involved			
			
*Median **(min- max)***	2.00 (1 - 8)	2.00 (1 - 5)	1-2 vs > = 3 P = 0.444
			Mann-Whitney U = 2142.500 P = 0.867
Prior therapy			
Paclitaxel	8 (11.9)	7 (10.8)	P = 0.109
Response to prior therapy			CR+PR vs SD+PD P = 0.799
*CR*	1 (1.5)	1 (1.5)	
*PR*	7 (10.4)	8 (12.3)	
*SD*	18 (26.9)	19 (29.2)	
*PD*	41 (61.2)	37 (56.9)	

### Compliance with the treatment

A total of 228 and 196 chemotherapy cycles were administered in the DC and D arms respectively; the median number of cycles received per patient was 3.0 (range 1-6) in the DC arm and 2.0 (range 1-6) in the D arm. The median duration of cycle was 31 days for the DC arm (range, 27-56 days) and 29 days for the D arm (range 27-47 days). Treatment administration was delayed in 61 (26.8%) DC and in 23 (11.7%) D cycles (p < 0.001). Twenty eight (45.9%) DC and 5 (21.7%) D cycles were delayed due to toxicity (both haematological and non-hematological) (p-value = 0.010). All other cycles were delayed for reasons not related to treatment or toxicity (i.e. patient's request for personal reasons, pending imaging studies for response assessment). Dose reductions were required in 41 (18.0%) DC and in 29 (14.8%) D cycles (p-value = 0.432). Dose reductions in both groups were mainly due to haematological toxicity. The median dose intensity for each drug were 22.1 mg/m^2^/week (range: 11.5-25.0 mg/m^2^/week) for docetaxel (88.2% of planned dose) and 87.1 mg/m^2^/week (range: 42.4-153.3 mg/m^2^/week) for carboplatin for the DC arm; in the docetaxel single-agent arm, median dose intensity for docetaxel was 23.3 mg/m^2^/week (range: 12.3-25.0 mg/m^2^/week; 93.3% of planned dose). There was no difference in the percentage of patients who received third-line treatment between the two arms (DC vs D: 59.7% vs 47.7%; p = 0.222) (Table 2).

**Table 2:** Third-line treatment per study arm

	DC (%)	D (%)	p-value
No treatment	27 (40.3%)	34 (52.3%)	0.222
Chemotherapy	28 (41.8%)	28 (43.1%)	0.999
EGFR-TKIs*	12 (17.9%)	3 (4.6%)	0.026

*Epidermal Growth Factor Receptor Tyrosine Kinase Inhibitor

### Response to treatment

Overall response rate was similar between the two arms (DC vs D: 10.4% vs 7.7%; p = 0.764). No complete responses were observed in both arms, while seven (10.4%) and five (7.7%) patients achieved a partial response (PR), in the DC and D arms respectively (Table 3). More patients in the single-agent arm had progressive disease (PD) when compared to combination arm; PD was observed in 46 (68.7%) and 51 (78.5%) patients, respectively (p = 0.239). The median duration of response was 4.0 months for the DC arm and 3.33 months for the D arm (p = 0.952).

**Table 3:** Median TTP and OS by treatment arm.

	**Treatment Group**		
		
	**DC** **(n = 67)**	**D** **(n = 65)**	
		
**Median PFS (mo)**	3.33	2.60	P = 0.012 (Log-Rank test)
*Min - Max*	0.2 - 23.0	0.5 - 17.8	
*95% CI*	(2.59 - 4.07)	(1.74 - 3.46)	
			
**1-year with no progression**	11.6%	5.4%	
			
**Median survival (months)**	10.27	7.70	P = 0.550 (Log-Rank test)
*Min - Max*	0.7 - 36.8	0.5 - 43.2	
*95% CI*	(7.07 - 13.47)	(3.39 - 12.01)	
			
**1-year survival Kaplan-Meier estimate (%)**	43.8%	40.3%	

**Figure 1:**
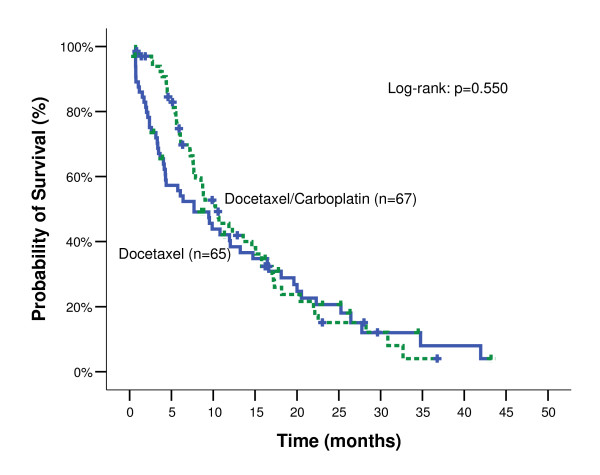
**Kaplan-Meier TTP curves by treatment arm**.

**Figure 2:**
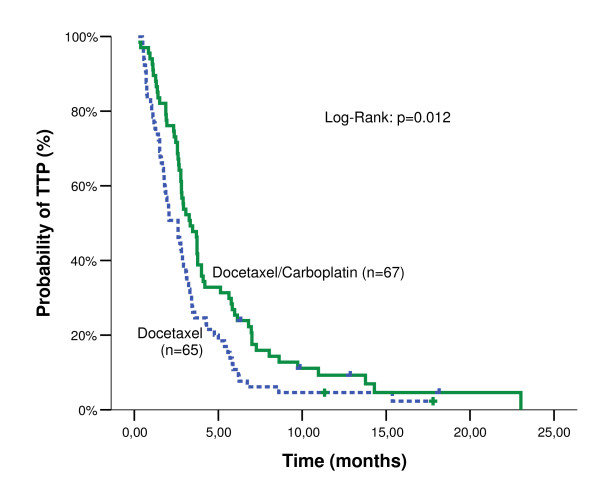
**Kaplan-Meier survival curves by treatment arm**.

After a median follow-up time of 28.0 months (min-max: 20.3-35.7) for DC arm and 34.5 months (min-max:22.1-46.9) for D arm (p-value = 0.425), the median PFS was 3.33 months (range: 0.2-23.0 months; 95% CI: 2.59-4.07) and 2.60 months (range: 0.5-17.8 months; 95% CI: 1.74-3.46) (p-value = 0.012; Figure 1) for the two arms, respectively (Table 3). Ιn addition, the median OS was 10.27 months for the DC arm (95% CI: 7.07-13.47) and 7.70 months for D arm (95% CI: 3.39-12.01) (Log-Rank test, p-value = 0.550) (Figure 2). The estimated 1-year survival rate was 43.8% and 40.3% for the DC and D arms, respectively. There was no difference in terms of ORR, PFS or OS between patients who received paclitaxel in first line treatment and those who did not, in both groups.

Forty (59.7%) patients in the DC arm and 31 (47.7%) in the D arm received third-line treatment (p = 0.222). The majority of patients in both arms received single-agent chemotherapy. However, there was an imbalance in the number of patients who received an EGFR tyrosine kinase inhibitor as third-line treatment (Table 2).

### Toxicity

Toxicity was assessed in all chemotherapy cycles and in all patients. Chemotherapy was in general well-tolerated and grade III/IV toxicities were relatively infrequent. No toxic deaths were observed. Table 4 summarizes all treatment-related toxicities. No significant differences were observed between the two arms in terms of hematological toxicity, with the exception of thrombocytopenia which was more pronounced in the DC arm; however, in the majority of cases it was of grade I and II and only two patients in the DC arm experienced grade III thrombocytopenia. All events resolved without any sequel. The most frequent grade III/IV hematological toxicity was neutropenia which occurred in 11 (16.4%) patients in the DC arm and four (7.5%) patients in the D arm (p = 0.098). In terms of non-hematological toxicity, mucositis (p = 0.027) and fatigue (p = 0.015) were more frequent in the D arm; in most patients these toxicities were of grade I or/and II. No differences were observed in post study toxic-deaths or other treatment-related serious adverse-events between the two arms.

**Table 4:** Haematological & Non-haematological toxicity per treatment arm

	**DC** **(n = 67)**		**D** **(n = 65)**		**DC** **(n = 67)**		**D** **(n = 65)**		**P-value**
	
	**GrIII**		**GrIII**		**GrIV**		**GrIV**		
	
	**N**	**%**	**N**	**%**	**N**	**%**	**N**	**%**	
	
Neutropenia	7	10.4	3	4.6	4	6.0	1	1.5	0.098
Anaemia	3	4.5	-	-	-	-	-	-	0.791
Thrombocytopenia	2	3.0	-	-	-	-	-	-	**< 0.001**
Febrile neutropenia	-	-	-	-	-	-	-	-	
*Nausea*	-	-	-	-	-	-	-	-	0.128
*Vomiting*	-	-	-	-	-	-	-	-	0.999
*Diarrhoea*	2	3.0	1	1.5	1	1.5	-	-	0.999
*Stomatitis*	-	-	-	-	-	-	-	-	0.027
*Constipation*	-	-	-	-	-	-	-	-	0.274
*Neuromuscular*	-	-	-	-	-	-	-	-	0.999
*Neurosensory*	-	-	-	-	-	-	-	-	0.678
*Allergy*	2	3.0	-	-	-	-	1	1.6	0.680
*Fatigue*	-	-	6	9.2	-	-	1	1.5	0.015
*Oedema*	-	-	-	-	-	-	-	-	0.680
*Other*									0.999

P-value for Neutropenia: for grade III/IV vs 0-II between the two groups

P-value for Anaemia: for grade II-IV vs 0/I between the two groups

P-value for Thrombocytopenia: for any grade vs grade O between the two groups

## Discussion and conclusions

Single agent docetaxel, or pemetrexed or EFGR TKIs (erlotinib and gefitinib) represent the standard of care for second-line treatment for NSCLC patients. However, the results observed with second-line treatment are generally poor with response rate of less than 10% and overall survival of 7-8 months [10-14]. One logical approach to improve these results is to evaluate combination regimens. This is the first phase III trial, at least in our knowledge, comparing docetaxel monotherapy with a docetaxel/carboplatin doublet in patients with recurrent or relapsed NSCLC.

The results of the current study demonstrate that second-line combination treatment with docetaxel/carboplatin offers a statistically significant therapeutic benefit compared to docetaxel monotherapy, in terms of PFS in patients with NSCLC who were not previously treated with docetaxel-based chemotherapy. Indeed, PFS was significantly prolonged in the DC arm as compared to D arm (p = 0.012). However, although there was a difference in terms of PFS this could not be translated to a significant difference in terms of OS (p = 0.550). Additionally, no difference was observed in ORR between the two treatment arms (p = 0.764). Taken together, these observations suggest that the higher percentage of patients with stable disease in the DC arm might have played a role in the differences observed in PFS.

Efficacy results in the current trial were obtained with an acceptable toxicity profile. With the exception of thrombocytopenia, the DC arm was not associated with higher toxicity. Indeed, both chemotherapy regimens were well tolerated, with toxicities being relatively infrequent and mild in the vast majority of cases.

The results observed in the single-agent docetaxel arm of the current study are remarkably similar to those observed in the phase III trials that led to registration of docetaxel as second-line treatment in NSCLC [10,11]. Indeed, OS in these trials was 5.7-7.0 months, median PFS 2.12-2.65 months, and ORR 5.8%-6.7%. The current study reported a median OS of 7.70 months, a median PFS of 2.60 months and an ORR of 7.7%. On the other hand, patients in the docetaxel-carboplatin arm had a longer OS (10.27 months) when compared to historical data [15,16]. It is noteworthy that the results of the current study are in accordance with the results of a phase III trial [24] and several randomised phase II trials comparing single-agent versus combination regimens as second-line treatment of NSCLC [25-28]. Although all these trials demonstrated a statistically significant improvement in PFS with chemotherapy combinations, this improvement in PFS was not translated into an OS prolongation. This observation should be attributed to the fact that an important proportion of patients with advanced/metastatic NSCLC have a good performance status and a acceptable expectancy of life making them suitable to receive third-line treatment; in the current study 71 (54%) out of 132 studied patients received third-line chemotherapy or TKIs.

The results of the current study are also in agreement with the results of a recently published meta-analysis based on individual data of 847 patients. Di Maio et al, compared the efficacy of a doublet chemotherapy regimen with single agent treatment as second-line treatment [29]. The conclusion of this meta-analysis was that although combination treatment was associated with significantly higher ORR and significant prolongation of TTP, this difference was not translated into a significant survival benefit [29]. Additionally, patients receiving combination treatment experienced significantly more toxicity. So, based on the lack of a survival benefit and the issue of toxicity, someone could argue against the use of combination regimens in the second-line setting. Furthermore, since patients' quality of life is of particular relevance especially for second-line treatment there is a need for a regimen with a favourable toxicity profile. On that basis we selected the bi-weekly mode of docetaxel administration because it has been reported that it is associated with a marked reduction in haematological toxicity [20].

Another important issue is the timing of administration of second-line treatment. A recently reported phase III study [30] demonstrated a statistically significant improvement in PFS and a non-statistically significant increase in OS when docetaxel was administered immediately after front-line gemcitabine/carboplatin doublet compared to its administration at the time of clinical disease progression; in addition, this strategy was not associated with increasing toxicity or decreasing QOL. Practically similar results were obtained with pemetrexed; indeed, in a recent phase III study, patients without disease progression after four cycles of platinum-based front-line treatment who received pemetrexed as maintenance treatment experienced a significantly longer median PFS and OS compared to patients who received placebo [31]. Additionally, erlotinib as maintenance treatment after front-line platinum based chemotherapy resulted in a significant prolongation of PFS [32].

Another controversial issue is the substitution of carboplatin for cisplatin and it could be argued our decision to use carboplatin instead of cisplatin. It is known that cisplatin-based doublets offer a small survival benefit as front-line treatment, when compared with carboplatin-based doublets [33]. On the other hand, carboplatin has a more favorable toxicity profile when compared with cisplatin [33]. Additionally, the administration of cisplatin requires the need for hydration and it is relatively contraindicated in patients with cardiopulmonary co-morbidities and renal insufficiency. Furthermore, given the practical advantage of carboplatin in terms of ease of administration, it could be argued that the small benefit achieved with cisplatin relative to carboplatin does not justify its use in clinical practice.

Another important issue is whether this study is clinically relevant, since many patients receive platinum-based first-line treatment. However, it should be noted that no phase III trial has clearly demonstrated that platinum-based doublets offer a survival benefit over platinum-free regimens. Furthermore, a recently published meta-analysis failed to show any difference in favor of platinum-based doublets when compared with platinum-free third generation combinations [17]. Thus, a number of NSCLC patients could receive platinum-free regimens as first-line treatment and this trial is relevant for these patients who could receive platinum derivatives as second line.

In conclusion, the combination of docetaxel/carboplatin was well tolerated as second-line treatment for NSCLC patients. Although the primary end-point of the study (prolongation of OS) could not be achieved, the docetaxel/carboplatin combination was associated with a significant clinical benefit in terms of PFS. Based on the results of the current study, docetaxel single-agent should be considered as one of the "standards" options for second-line treatment of advanced/metastatic NSCLC while its combination with carboplatin might be administered in patients with good PS who were not previously treated with a platinum-based doublet or within a well designed clinical trial. Further research is required for the development of more effective chemotherapeutic regimens and for the integration of newer targeted agents into NSCLC treatment.

## Competing interests

The authors declare that they have no competing interests.

## Authors' contributions

AGP, SA and VG have made substantial contribution to conception and design, interpretation of data and been involved in drafting the manuscript. AGP and SA were involved in manuscript writing. AA, IV, KS, NK, GP, AK, and VG enrolled patients to study protocol. EK has made substantial contribution acquisition of data; interpretation of data. All authors read and approved the final manuscript

## Pre-publication history

The pre-publication history for this paper can be accessed here:

http://www.biomedcentral.com/1471-2407/10/633/prepub
